# Adjusting Brain Dynamics in Schizophrenia by Means of Perceptual and Cognitive Training

**DOI:** 10.1371/journal.pone.0039051

**Published:** 2012-07-18

**Authors:** Tzvetan Popov, Brigitte Rockstroh, Nathan Weisz, Thomas Elbert, Gregory A. Miller

**Affiliations:** 1 Department of Psychology, University of Konstanz, Konstanz, Germany; 2 Department of Psychology, University of Delaware, Newark, Delaware, United States of America; 3 Department of Psychology, University of Illinois at Urbana-Champaign, Champaign, Illinois, United States of America; University of Granada, Spain

## Abstract

**Background:**

In a previous report we showed that cognitive training fostering auditory-verbal discrimination and working memory normalized magnetoencephalographic (MEG) M50 gating ratio in schizophrenia patients. The present analysis addressed whether training effects on M50 ratio and task performance are mediated by changes in brain oscillatory activity. Such evidence should improve understanding of the role of oscillatory activity in phenomena such as M50 ratio, the role of dysfunctional oscillatory activity in processing abnormalities in schizophrenia, and mechanisms of action of cognitive training.

**Methodology/Principal Findings:**

Time-locked and non-time-locked oscillatory activity was measured together with M50 ratio in a paired-click design before and after a 4-week training of 36 patients randomly assigned to specific cognitive exercises (CE) or standard (comparison) cognitive training (CP). Patient data were compared to those of 15 healthy controls who participated in two MEG measurements 4 weeks apart without training. Training led to more time-locked gamma-band response and more non-time-locked alpha-band desynchronization, moreso after CE than after CP. Only after CE, increased alpha desynchronization was associated with normalized M50 ratio and with improved verbal memory performance. Thus, both types of cognitive training normalized gamma activity, associated with improved stimulus encoding. More targeted training of auditory-verbal discrimination and memory additionally normalized alpha desynchronization, associated with improved elaborative processing. The latter presumably contributes to improved auditory gating and cognitive function.

**Conclusions/Significance:**

Results suggest that dysfunctional interplay of ocillatory activity that may contribute to auditory processing disruption in schizophrenia can be modified by targeted training.

## Introduction

Cognitive deficits are a core feature of schizophrenia, and cognitive remediation is an increasingly prominent goal of rehabilitation programs [1]. Training effects are usually evaluated via performance on neuropsychological tests, global function, or symptom severity. Cognitive processes are associated with cortical function, thus cognitive deficits with abnormal cortical mechanisms. Hence, cognitive training effects should be evident in cortical and behavioral measures. Indeed, training procedures facilitating neuroplasticity (e.g., massed practice, shaping, and reinforcement) have been shown to improve verbal learning and memory together with changes in cortical correlates of auditory discrimination accuracy [2]–[5]. Cognitive training increased activation in prefrontal networks related to attention and working memory [6], and cognitive remediation therapy predicted preservation of gray-matter volume over two years in hippocampal, parahippocampal, fusiform, and amygdala regions [7].

Oscillations in neural activity are believed to be critical in mechanisms of normal and abnormal stimulus encoding, recall, and perceptual and cognitive performance [8]–[12]. Such oscillations are complementary to conventional EEG event-related brain potentials (ERPs) or their MEG analogs, event-related fields (ERFs), representing more discrete neural events. This has been demonstrated for an abnormal ERP phenomenon in schizophrenia: P50 and its magnetic analog M50 are related to the attenuation of the response to a second of two brief click stimuli presented in pairs. The amplitude ratio (S2-evoked/S1-evoked) serves as a measure of auditory sensory gating, with a high ratio suggesting impaired sensory gating in schizophrenia [13], [14]. Analysis of M50 together with time-locked and non-time-locked oscillatory activity in the paired-click design showed that patients’ larger M50 gating ratio was associated with less non-time-locked fronto-parietal desynchronization in the 10–15 Hz frequency band starting 200 ms before the onset of S2 [15]. Patients also produced smaller alpha (8–12 Hz) and gamma (60–80 Hz) responses to the first stimulus. Results further suggested that the deviant M50 gating ratio in schizophrenia results from an altered interplay of these processes. This finding adds to the growing literature demonstrating oscillatory abnormalities in schizophrenia in relation to dysfunctional information processing (e.g. [16]–[19]) and their implications for neuroplasticity [20], [21].

Exploration of abnormal oscillatory activity in schizophrenia is potentially valuable both to identify brain mechanisms altered by psychological intervention such as cognitive training and to address a debate about whether deficient sensory gating in schizophrenia is due to an abnormal initial response to S1 or to a failure to attenuate the response associated with S2 [22]. We reported [2] that deficient sensory gating was due to exaggerated S2 responses, which were sensitive to an appropriate training intervention. That analysis was based on brain electromagnetic source reconstruction using two equivalent current dipoles under the assumption that early (<100 ms) auditory cortex activity originates mainly from bilateral superior-temporal gyri (STG). Subsequently [15] we applied a more data-driven, exploratory analysis, taking into account both inter-trial variability and differences in various frequency bands. Whereas the differences in sensory gating ratio were confined to abnormal S2 dipole strength, the single-trial spectral analysis revealed group differences in brain activity following both S1 and S2.

The respective roles of S1 and S2 abnormalities in the gating ratio, both its exaggeration in schizophrenia and its normalization with cognitive training, remain to be determined. With this perspective, the present analyses focused on functional changes in oscillatory activity at high temporal resolution with respect to S1 and S2, which can only be addressed by single-trial sensor-space analyses rather than cross-trial averages in sensor space or source space. Accordingly, the present report addresses whether changes in oscillatory activity, due to training that normalized M50 gating, point to S1 or S2 effects as the mechanism of this successful intervention. To this end, data from the sample described in [2] were analysed to examine the following hypotheses:

1. Cognitive training, in particular targeted training fostering auditory-verbal discrimination, increases (normalizes) both time-locked gamma (60–80 Hz) response time-locked to the first click and non-time-locked alpha response (8–12 Hz) around the second click in schizophrenia patients.
2. Training-induced changes in these oscillatory measures correlate with training-induced normalization of M50 ratio, supporting the claim that these oscillatory measures explain abnormal M50 ratio in schizophrenia.
3. Training-induced changes in these oscillatory measures predict verbal learning and memory performance, evidence that oscillatory activity reflects basic mechanisms contributing to cognitive dysfunction in schizophrenia.

## Results

**Figure 1:**
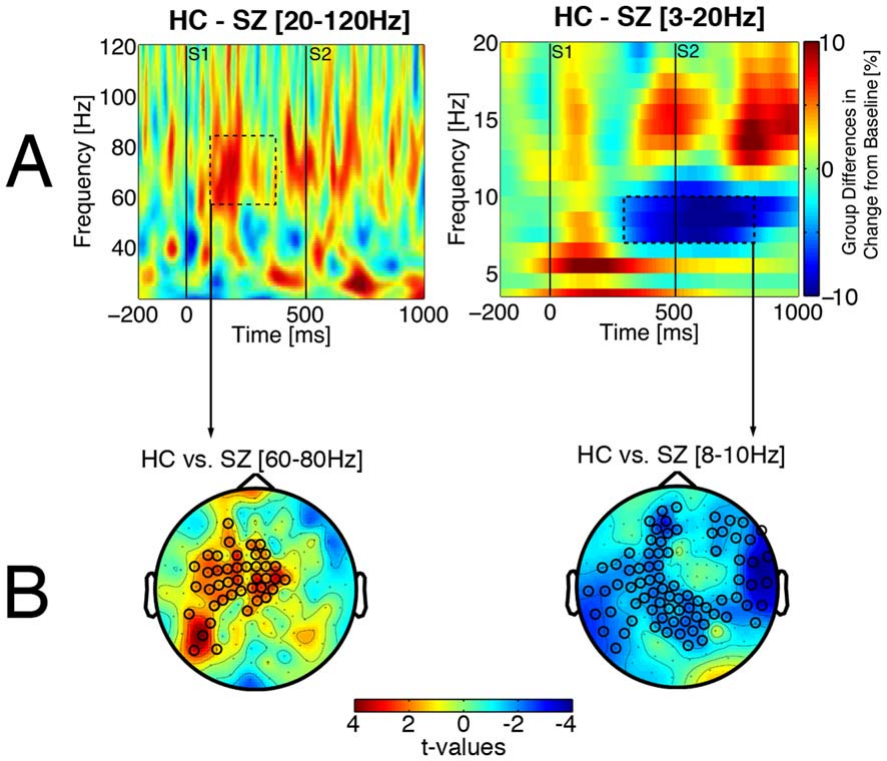
Group differences prior to training. Time-frequency plots showing group differences in change from pre-S1 baseline (A), averaged across respective sensor clusters in 1B. Red indicates larger power increase from pre-S1 baseline in controls than in schizophrenia patients, and blue indicates larger power decrease in controls than in patients. Dashed rectangles indicate time-frequency window of significant group differences. Color bar indicates percent change from pre-S1 baseline. B: Clusters of sensors showing significant group differences in gamma increase (left panel, 60–80 Hz, 100–400 ms after S1) and alpha decrease (right panel, 8–10 Hz, 300–800 ms after S1). Sensors belonging to a significant cluster marked with circles. Color bar indicates size of group difference represented in t-values.

Changes with training should be manifest in abnormal oscillatory phenomena. Therefore, permutation tests on pre-training data from the present sample were used to identify frequencies and time periods of interest for evaluation of the impact of training. Figure 1 illustrates two pre-training differences between patients and healthy controls, as previously reported for a slightly larger sample [15] from which the present sample was drawn: patients had less increase in time-locked gamma 100–400 ms after S1 onset and less decrease in non-time-locked alpha starting about 300 ms after S1 and continuing until about 300 ms after S2, thus 300–800 ms after S1 onset. These pre-training abnormalities were confirmed by cluster-based permutation tests for gamma (Figure 1B left panel, t_cluster_>2, p_cluster_ = .02) and alpha (Figure 1B right panel, t_cluster_<−2, p_cluster_ = .03).

**Figure 2:**
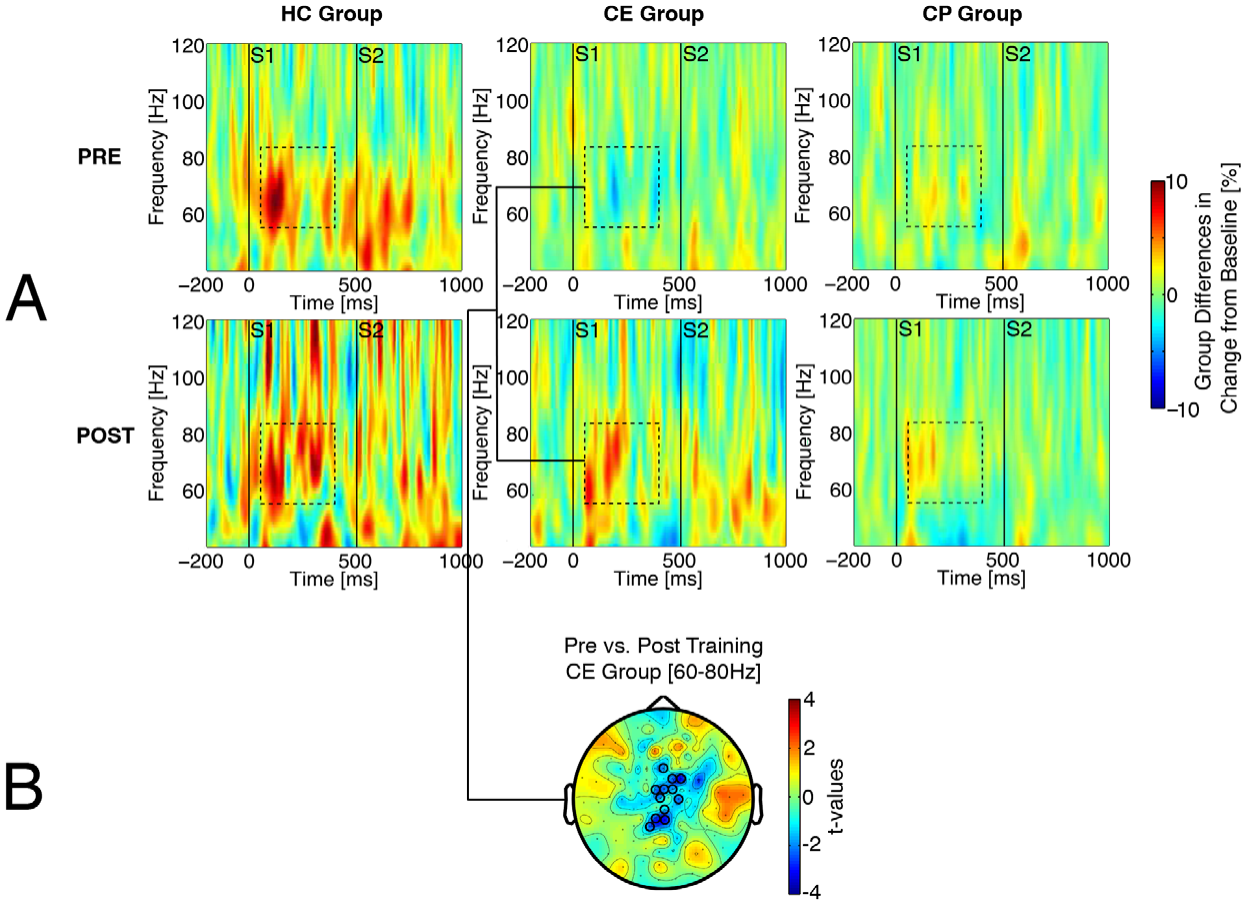
Time-frequency plots illustrating a training effect on gamma power. Panel A depicts an increase 100–400 ms after S1 onset (dashed squares) before vs. after training, averaged across the sensor cluster defined in 2B. B: Sensor cluster showing significant CE training effect on gamma power increase defined by cluster-based permutation analysis.

**Figure 3:**
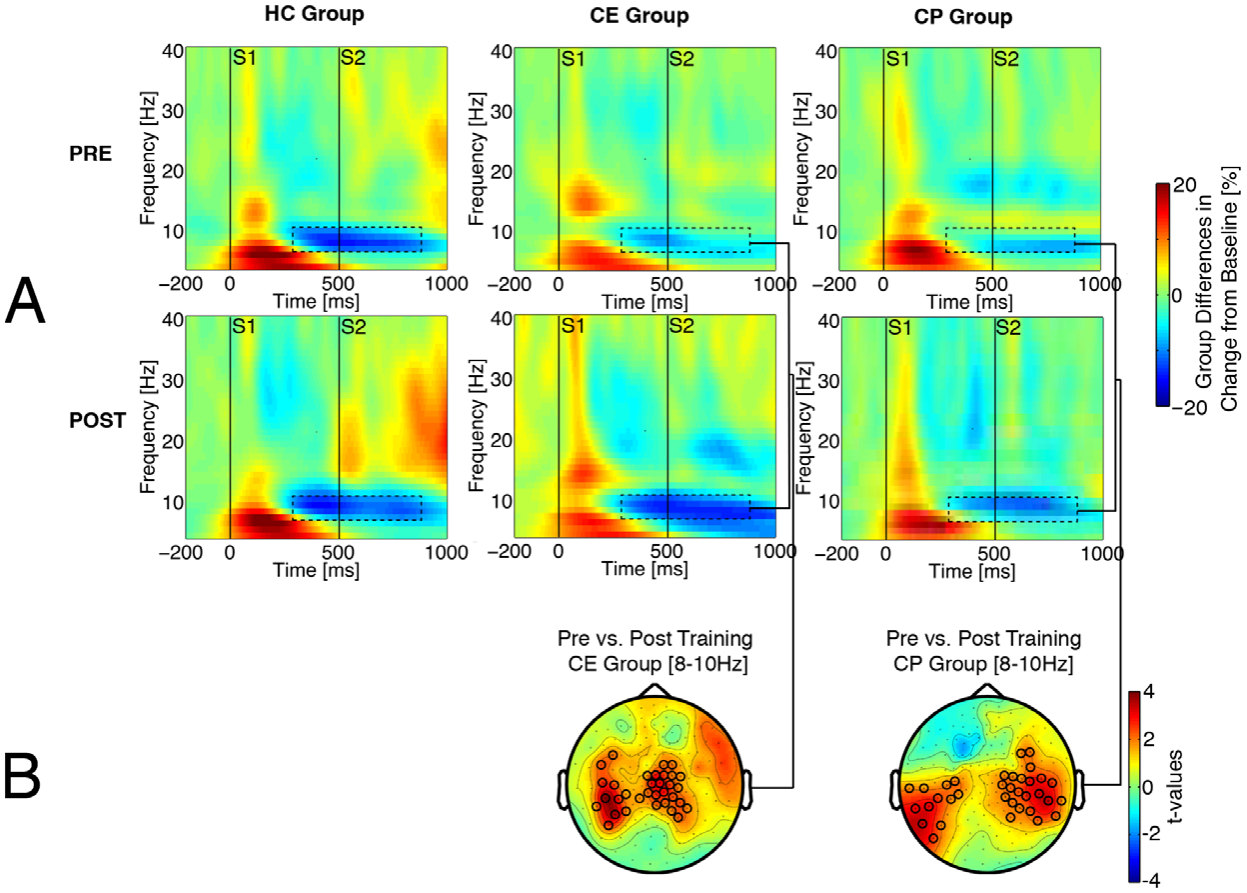
Time-frequency plots illustrating a training effect on alpha power. Panel A depicts a decrease around S2 (300–800 ms after S1, dashed rectangles) before vs. after training, averaged across respective clusters defined in 3B. B: Clusters of significant training effects displayed separately for CE and CP patient groups, scored for the ROIs defined after cluster-based permutation analysis.

Based on these pre-training findings, which identified frequencies and time periods of interest, permutation tests identified clusters of MEG sensors for analysis of training effects. Figures 2 and 3 illustrate training effects on time-locked gamma and non-time-locked alpha, respectively. Activity in the region identified for time-locked gamma responses (Figure 2B) and the region identified for non-time-locked alpha responses (Figure 3B) was submitted to a Group (CE/CP/HC)×Time (session 1/session 2)×Band (gamma/alpha) ANOVA. Time is complicated by the HC group not receiving treatment, and Band is driven by characteristically different activity scales for gamma and alpha. Thus, Time and Band effects in these omnibus ANOVAs were of no interest. A Time×Band interaction (F(1,48) = 27.5, p<.001) reflected a decrease in alpha activity vs. an increase in gamma activity at retesting.

The relevant omnibus test for hypothesis 1 is the Group×Time×Band interaction (F(2,48) = 6.0, p = .005), which was explored with more specific tests. First, training effects were verified in the patient groups via simple main effects of Time for time-locked gamma (F(1,34) = 5.1, p<.03) and non-time-locked alpha (F(1,34) = 24.9, p<.001). The HC group showed no significant test/retest effects for gamma or alpha.

Figure 2 illustrates how type of training modulated time-locked gamma response. Permutation tests confirmed changes in a centro-parietal sensor cluster after CE (t_cluster_ >2, p_cluster_<.03) but not after CP (Figure 2B). A Group×Time ANOVA yielded Group (F(2,48) = 8.8, p<.001) and marginal Time (F(2,48) = 2.7, p<.11) effects. In exploratory tests in light of the omnibus 3-way interaction, the CE group improved over time (F(1,19) = 6.4, p<.02), but the CP group did not (F<1). In addition, patients’ pre-training gamma abnormalities (CE vs. HC: t(33) = −3.5, p<.001; CP vs. HC: t(29) = −2.7, p<.01) disappeared in the CE group (t(33) = −1.2, p = .24). This was less clear in the CP group (t(29) = −1.7, p = .09).

Figure 3 illustrates how type of training modulated non-time-locked alpha decrease beginning prior to S2 (Figure 3A), with larger alpha decrease after CE (middle panels) than after CP (right panels). Cluster-based permutation tests confirmed the change after CE for bilateral temporal and central sensor clusters (t >2, p_cluster_<.02) and after CP for a left temporal cluster (p_cluster_<.05) and a trend for a right temporal cluster (p_cluster_ = .07). The ANOVA confirmed training changes for both groups (Group×Time, F<1; CE Time F(1,19) = 28.6, p<.001; CP Time F(1,15) = 5.5, p<.03). Comparing post-training activity, there was less alpha after CE than after CP (t(34) = −2.01, p = .05). For both patient groups, pre-training abnormalities in alpha power (CE vs. HC, t(33) = 2.5, p<.02; CP vs. HC, t(29) = 1.9, p = .07) disappeared after training (CE vs. HC, t(33) = −1.4, p = .17; CP vs. HC, t(29)<1; Group×Time, F(2,48) = 3.8, p<.05).

Although training prompted changes in both frequency bands, no correlation between the changes in time-locked gamma response following S1 and the changes in non-time-locked alpha power decrease starting before S2 were evident in either group (CE: r = .27, p = .24; CP: r = .25, p = .39). Thus, changes in the two frequency bands were distinct. Patients in the CE group demonstrated large training effect sizes (Hedges’ g) in both measures (gamma increase g = −.81, SE = .33 CI [−1.46: −.17], alpha decrease g = 1.28, SE = .35 CI [.6: 1.96]). Patients in the CP group demonstrated small to medium effects (gamma increase: g = −.35, SE = .36 CI [−1.05∶ 0.35], alpha decrease: g = .49, SE = .36 CI [−.21∶ 1.2]).

**Figure 4:**
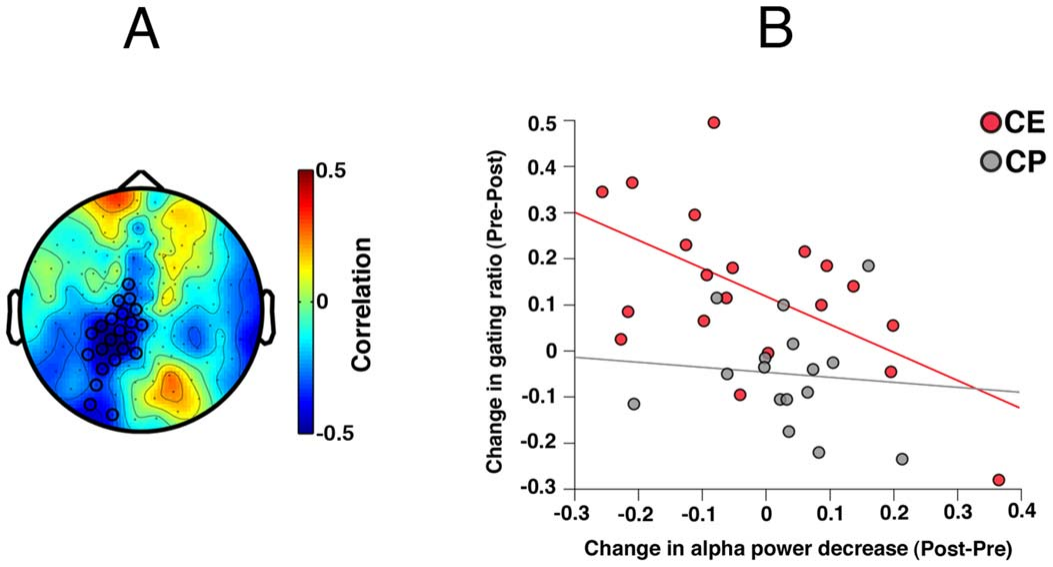
Topography of the relationship between the change in alpha power decrease and the change in M50 gating ratio. Panel A illustrates significant cluster of MEG sensors (p_cluster_<0.05) obtained after correction for multiple comparisons. Sensors belonging to the significant cluster are marked with black circles. Color bar indicates range of correlation coefficients. B: Relationship between change in alpha power decrease (abscissa, negative values indicate improvement) and pre-to-post-training change in M50 gating ratio (ordinate, positive values indicate changes towards normal, that is, smaller gating ratio) by patient training group based on the ROI in 4A.

The second hypothesis addressed whether training effects on M50 gating ratio [2] were mediated by changes in oscillatory activities. This should be evident in the relationship between changes in oscillatory activity and changes in M50 ratio. As reported in [2], M50 ratio normalized after CE and not after CP (Group×Time F(1,37) = 11.97, p<.002). In support of the second hypothesis, Figure 4A illustrates a negative correlation between M50 gating ratio and non-time-locked alpha decrease in the centro-parietal cluster (p_cluster_<.05). Figure 4B shows that the change in M50 ratio varied with the change in alpha decrease in the CE group (Pearson r = −.57, p<.01, Spearman r = −.46, p<.05), not in the CP group. However, a test for homogeneity of regression slopes did not confirm a significant difference of correlations. No significant relationships between the training-induced changes in time-locked gamma-band response and the change in M50 ratio were evident.

**Figure 5:**
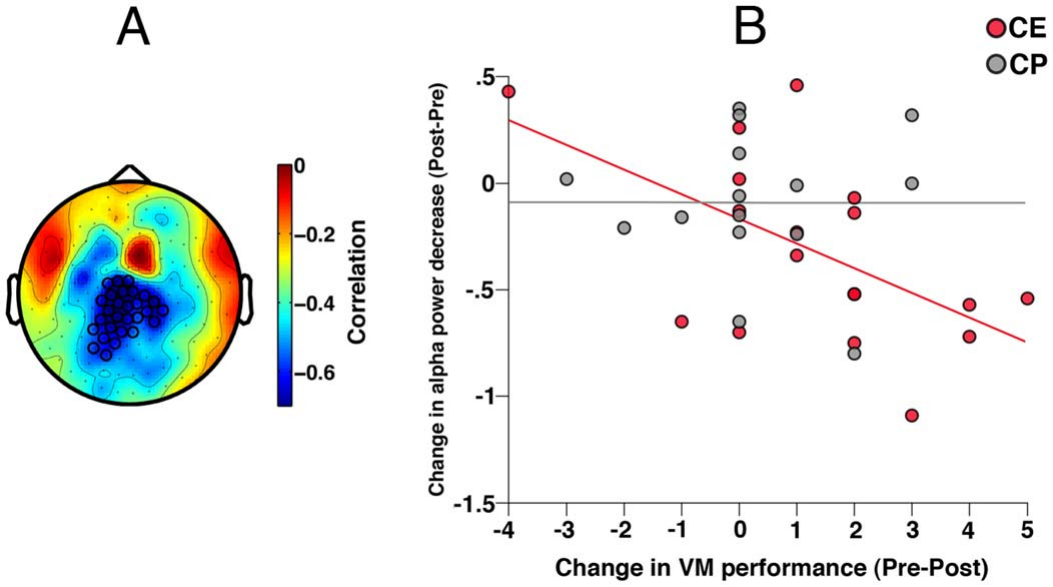
Topography of the relationship between verbal memory performance and alpha power decrease. Panel **A** illustrates significant cluster of MEG sensors (p_cluster_<0.01) obtained after correction for multiple comparisons. Sensors belonging to the significant cluster are marked with black circles. Color bar indicates range of correlation coefficients. **B**: Relationship between change in verbal memory (VM) performance (abscissa, positive values indicate more improvement) and pre-to-post-training change in alpha power decrease (ordinate, negative values indicate more alpha power decrease, thus, improvement) by patient training group based on the ROI in 5A.

**Figure 6:**
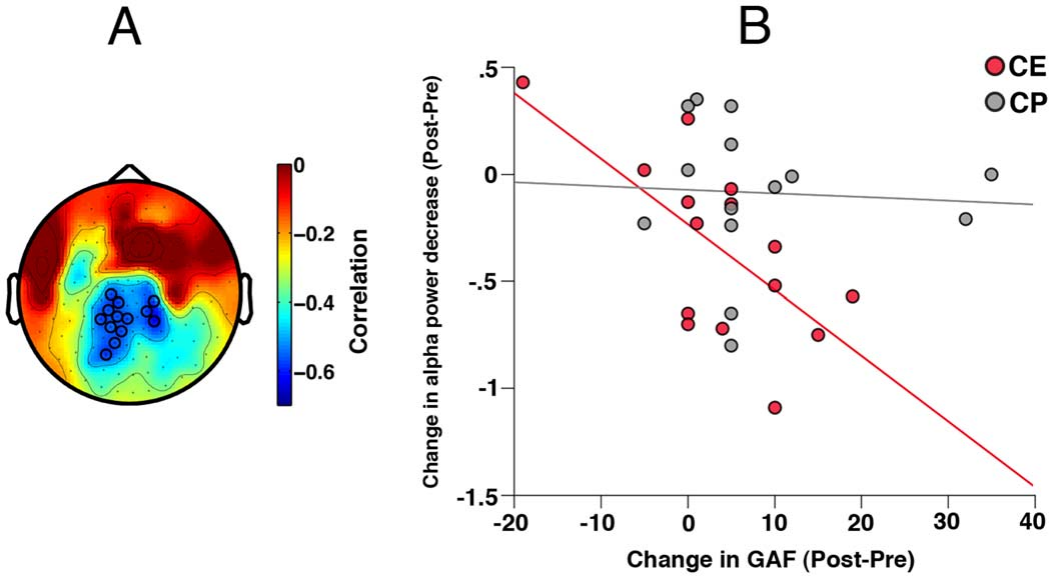
Topography of the relationship between global function rating and alpha power. Panel **A** illustrates significant cluster of MEG sensors (p_cluster_<0.02) obtained after correction for multiple comparisons. Sensors in the cluster are marked with black circles. Color bar indicates range of correlation coefficients. **B**: Relationship between change in Global Assessment of Functioning (GAF, abscissa: positive values indicate improvement) and pre-to-post-training change of alpha power decrease (ordinate: negative values indicate more alpha power decrease, thus improvement) by patient training group based on the ROI in A.

The third hypothesis addressed the relationship between changes in the two oscillatory measures, gamma increase and alpha decrease, and changes in cognitive performance*:* As reported in [2], participants in CE displayed more improvement in verbal memory than did participants in CP (Time F(1,30) = 10.83, p<.01; Group×Time F(1,30) = 5.64, p<.03; CE Time t(16) = 4.1, p<.001; CP Time t(14)<1). Correlating performance measures with training-induced changes in oscillatory measures showed that improvement after CE was related to more change in alpha power decrease. Confirmed for a centro-parietal sensor cluster (p_cluster_<.01, Figure 5A), the change in alpha power decrease varied with verbal memory improvement (Figure 5B) in the CE group (Pearson r = −.56, p<.02, Spearman r = −.5, p<.04), not in the CP group (Pearson r<.01, Spearman r<.01). The difference in correlations between groups approached significance, F = 2.89, p = .09. Training-induced changes in alpha power decrease were also associated with improvement in Global Assessment of Functioning, confined mainly to change in oscillatory activity in a centro-parietal sensor cluster (p_cluster_<.02, Figure 6A) with CE (Pearson r = −.66, p<.01, Spearman r = −.48, p = .059, Figure 6B) but not with CP. The CE and CP correlations differed (F = 5.27, p<.05).

## Discussion

The present study sought to identify oscillatory abnormalities that may have carried cognitive treatment effects reported in a recent study of schizophrenia. Oscillations are believed to be a crucial element of neuroplasticity, as “oscillations provide a temporal structure that allows for precise alignment of the amplitude and temporal relations of presynaptic and postsynaptic activation that determine whether a strengthening or weakening of synaptic contacts occurs” p. 72–73 in [20], see also [19], [23]. Abnormalities in neuroplasticity supporting structural and functional reorganization as a consequence of learning and experience have been proposed to contribute to schizophrenia psychopathology [24]–[27], and TMS studies have provided evidence of impaired neuronal plasticity in schizophrenia [28]–[30]. Diverse factors contributing to dysfunctional neuroplasticity in schizophrenia have been proposed, including insufficient dopaminergic modulation of learning and reinforcement sensitivity [31]–[33], altered long-term potentiation [28], progressive gray matter loss [34], [35], anomalies in GABA-driven rhythm-generating networks [19], and glutamatergic/NMDA-mediated gamma synchrony deficits [21]. Reduced high-frequency oscillations in schizophrenia, including beta and gamma frequencies, have been proposed to be a consequence of abnormal neurodevelopment, with altered functional plasticity a crucial mediator [19], [20], [23]. The previous [15] and present analyses of single-trial neuromagnetic oscillations add to this discussion, as abnormalities in gamma response were confirmed. Even more important is previous [2] and present evidence of training-induced changes that suggest some neuroplastic potential in schizophrenia patients.

In present analyses, two oscillatory phenomena were significantly modified and somewhat normalized by cognitive training. Both phenomena have been related to abnormal brain dynamics in schizophrenia, lower-than-normal gamma response and alpha desynchronization. Effects of cognitive training on average ERPs [2]–[4] and measures of cortical plasticity [5], [6] have also been reported. Present analyses document modification of measures of single-trial brain dynamics.

Abnormalities and their normalization were demonstrated for gamma and alpha bands. Gamma oscillations are considered a measure of neural synchrony, crucial for the development of cortical networks [19], and abnormal gamma oscillations are thought to index neurodevelopmental pathology in schizophrenia [21], [23], [36]. The present finding of abnormal time-locked gamma response strengthens this view. Importantly, the abnormality can be modified, supporting a role of gamma oscillations in neuroplasticity [19], [20] and suggesting plasticity in schizophrenia, to be considered in rehabilitation programs.

Abnormalities in the alpha band in schizophrenia have been reported in terms of reduced frontal EEG alpha power [37] and reduced event-related alpha desynchronisation [38]–[40]. Alpha power decrease is considered an important element in the cross-frequency interplay of lower and higher frequencies [12], crucial for coordinated information flow to and processing in task-relevant areas and networks. An altered interplay of alpha and gamma in schizophrenia has been interpreted as sign of impaired recruitment of brain networks [41]. Present training methods permitted modification of this abnormal interplay by normalizing both oscillatory phenomena. The two changes were not correlated, indicating two distinct effects of training. Increased gamma synchrony time-locked to a first stimulus has been related to efficient repetition suppression, suggesting top-down influence on the processing of a subsequent stimulus [42]. Alpha power decrease, in this cross-frequency interaction, indicates increased excitability of an activated cortical area [42], [43], facilitating the processing of incoming input [44] and shaping neuronal network engagement and inhibition in task-irrelevant areas [12].

Effects were larger after a type of training derived from principles of neuroplasticity (massed practice, shaping, and reinforcement) that concentrates on signal discrimination accuracy in the auditory-verbal system than after a less focused training program. CE specifically trained auditory-verbal discrimination accuracy and emphasized working memory. Thus, training addressed psychological processes that have been related to oscillatory phenomena and reported to be deficient in schizophrenia, perceptual and cognitive processes such as stimulus encoding [9], [45], memory trace formation [8], [18], [45], [46] **,** top-down modulation of initial auditory information processing [47], [48], auditory recognition and retrieval processes [48], and activation of attention resources [44], [49], [50]. Hence, larger effects of CE than of CP on sensory gating (M50 ratio), overt performance (verbal memory), and non-time-locked alpha power decrease in a paired-click design requiring auditory memory trace formation and stimulus comparison presumably resulted from the more targeted training.

Although modification was demonstrated for both oscillatory phenomena, the relationship between oscillatory improvement, conventional ERFs, and behavioral variables was particularly prominent for alpha power decrease and only significant after CE. This suggests a prominent role of alpha activity in the gating of information flow and processing that may affect the processing of S2 in the paired-click design and thus M50 ratio [15]. Training effects on gamma activity (indicating improved S1 encoding) may have influenced training effects on the subsequent brain state, indicated by enhanced alpha power decrease and M50 ratio normalization, reflecting improved processing of the paired clicks. Present analyses thus support the hypothesis about oscillatory mechanisms contributing to sensory gating [15], [16].

Conclusions from the present analyses are constrained by methodological limitations. The primary goal, to identify functional changes in oscillatory activity with respect to S1 and S2, required sensor-space analyses because of the need to look at single-trial activity. Sensor-space analyses are subject to cross-talk and spatial smearing. If feasible with single-trial data, source analyses could be informative, as they provide one means of avoiding the cross-talk problem. Beneficial effects of gamma increase and alpha decrease should be evaluated with tasks, ERP/ERF components, and cognitive tests beyond those employed in the present study. Assessment of long-term stability of training effects on cognitive measures and global function and of training-induced changes in oscillatory phenomena is warranted to explore neuroplastic capacity in schizophrenia.

In summary, present results indicate that specifically designed cognitive training procedures affect oscillatory phenomena that are thought to reflect crucial dysfunctional neural network dynamics in schizophrenia. Furthermore, results indicate considerable potential for cortical reorganization in schizophrenia. If substantiated, the findings commend neuroplasticity-oriented training in the development of cognitive remediation procedures.

## Materials and Methods

### Ethics Statement

The clinical trial, the study protocol, and the recruitment procedure were approved by the ethics committee of the University of Konstanz. Patients were recruited from a ward overseen by the University, and this committee is also responsible for ethical approval of the recruitment of patients.

### Participants

Data were analyzed for 36 inpatients recruited, evaluated, and trained at the regional Center for Psychiatry as well as 15 healthy participants. The samples were drawn from the 39 patients and 18 healthy participants recruited for a study of M50 gating ratio described in [2]. As described in [2], patients were randomly assigned to one of two training protocols and completed 20 individual training sessions within 4 weeks. Healthy participants recruited to be comparable to the patient sample in age and gender participated in the MEG protocol twice separated by 4 weeks for assessment of stability of electromagnetic measures. Data from 3 patients and 3 controls were excluded from present analyses due to non-physiological artifacts, so the present report includes 36 patients and 15 controls (see Table 1 for demographic information). Groups did not differ in gender distribution, age, or handedness. As reported in detail in [2] patients were included if they met criteria for an ICD diagnosis of paranoid-hallucinatory schizophrenia (F20.0), age 20–50 years, normal intellectual function, and no history of neurological condition or disorder including epilepsy or head trauma with loss of consciousness.

**Table 1:** Demographic variables for schizophrenia patients (SZ) and health controls (HC).

	Age (M±SD)	Gender (m/f)	LQ (M±SD)	Education (M±SD)
**Healthy controls (HC, n = 15)**	29.9/7.9	14/1	86.3/37.2	16.3/2.3
**Patients (SZ, n = 36)**	30.2/7.9	32/4	60.7/64.2	12.7/2.5
**Stat. Difference**	t(49) = 0.1 ns.	Chi^2^ = 2.1 ns.	t(49) = −1.4 ns.	t(49) = −4.8**

Note: Variables (except gender) are expressed in mean and standard deviation. LQ = Handedness lateralization quotient. Statistical effects are listed in the bottom row, *p<.05, **p<.01.

Patients meeting inclusion criteria were informed about the training and measurement protocol and were included in the MEG assessments and random assignment protocol after signing written informed consent. The training study was approved as a clinical study (Clinical Trials Registration Number: ClinicalTrials.gov: Training-Induced Cerebral Reorganization in Schizophrenia; http://www.clinicaltrials.gov/; NCT00695708). Patients participated either in a computerized training that implemented principles of neuroplasticity and specifically targeted auditory-verbal discrimination accuracy and working memory (Cognitive Exercise, *CE*, adapted from a protocol of Posit Science, San Francisco, USA) or in a computerized, broad-spectrum cognitive training protocol of similar duration and intensity widely used in Germany (Cognitive Package, *CP*, Cogpack; Marker Software, Ladenburg, Germany). CE consists of six computerized exercises: judging gradually more difficult distinctions between frequency modulation sweeps of auditory stimuli increasing or decreasing in frequency, distinguishing phonemes in synthesized speech, identifying arrays of open and closed syllables in spatial and temporal context, discriminating tone frequencies, and remembering details of a short narrative. CP includes a much broader array of 64 exercises of visuomotor skills, vigilance, comprehension, language, memory, logic, and everyday skills. Each CP exercise is available with several variations. Both training protocols were computer-based and adaptive to foster positive reinforcement and avoid failures. Treatment methods were similar with respect to total duration of treatment (4 weeks). CE comprised 60-min daily sessions on 20 consecutive workdays, whereas CP followed the standard protocol as recommended by the developers: a series of tasks to be accomplished during each of three 60–90 min sessions/week. Treatments were broadly similar in frequency and duration of training sessions and in observed participant effort and tolerance.

Cognitive test performance and global function were assessed in patients before and after training. Scores for verbal memory were obtained from the German version of the California Verbal Learning Test [51]. Global function was assessed by each patient’s psychiatrist or psychologist in charge based on the (DSM-IV) Global Assessment of Functioning Scale. Psychiatrists were blind to the assignment of patients to the training (CE or CP), as were assistants accomplishing the cognitive testing. Moreover, a subset of the MEG data was analyzed in parallel by an assistant blind to the assignment of patients to the training, in order to avoid bias.

### Data Acquisition and Analysis

In the paired-click design, 100 pairs of 3 ms square-wave clicks were presented with a 500 ms onset-to-onset inter-stimulus interval and a varied inter-trial interval (offset to onset 7–9 sec). Clicks were presented at 50 dB above subjective hearing level, determined separately for each ear, and delivered via non-ferromagnetic tubes. No task was involved, except that participants were asked to attend to a small fixation point throughout the recording. MEG was recorded while participants lay on their back, using a 148-channel magnetometer (MAGNES™ 2500 WH, 4D Neuroimaging, San Diego, USA). Data were continuously recorded with a sampling rate of 678.17 Hz and a bandpass filter of 0.1 to 200 Hz. The subject’s nasion, left and right ear canal, and head shape were digitized with a Polhemus 3Space® Fasttrack prior to each session. Epochs of 1000 ms before and 2000 ms following S1 were identified from the continuous recordings. Trials with large artifacts (e.g., channel jumps) were rejected, and independent component analysis (ICA) was performed in order to identify and reject eye blink and muscle artifacts. Number of trials included in the analysis did not differ for patients and controls either pre- or post-treatment: pre-treatment CE: M = 84.5, SD = 11.5, CP: M = 84.5, SD = 11.2, HC: M = 84.6, SD = 10.6, post-treatment CE: M = 90.1, SD = 7.9, CP: M = 88.3, SD = 7.2, HC: M = 91.2, SD = 6.1 (all F<1). In [15] M50 ratio and its abnormality in schizophrenia patients were explained by smaller-than-normal alpha desynchronisation preceding S2, which accounted for insufficient S2 gating. The dynamic interplay of S1 encoding (reflected in time-locked gamma response) and preparation for S2 processing (reflected in non-time-locked alpha activity) was considered crucial for auditory gating (reflected in M50 ratio).

Time-locked activity, often also referred to as ‘evoked’, reflects brain activity consistently associated in latency and phase with the onset of a stimulus, apparent after averaging across trials. Non-time-locked activity, often referred to as ‘induced’, is estimated on a single-trial basis and reflects additional brain activity triggered by a stimulus but not strictly locked to its onset. Distinguishing time-locked and non-time-locked oscillatory activity prompts consideration of gating as a complex process, where early auditory cortex activity might be influenced by higher-order brain areas in a top-down manner. In fact, recent evidence demonstrates that expected vs. unexpected occurrence of the second auditory stimulus modifies both evoked time-locked activity and gamma-band synchrony [42].

Spectral analysis was performed according to the procedures described in [9]. Convolution with a complex Morlet wavelet was applied to single trials: *w(t, f_0_) = A*exp*(-t^2/^2σ_t_^2^)*exp*(2iπf_0_t)*, where *σ_t = _m/2πf_0_*, *i* was the imaginary unit, and *A = (σ_t_√π)^−1/2^* was the normalization factor. The trade-off between frequency and time resolution was determined by the constant *m* = 7 cycles per analysis, with resulting analysis bins e.g. 700 ms wide for 10 Hz and 70 ms wide for 100 Hz. (An analysis with a smaller constant *m*, thus providing better temporal resolution at the cost of poor frequency resolution, supported present findings and is provided in the supplement.) Time-frequency representation of power (TFR) was calculated by averaging the squared absolute values of the convolutions over trials. Using a 500 ms baseline preceding S1 onset, TFR of post-stimulus activity (postA) was expressed as change relative to pre-stimulus activity (preA): (postA–preA)/preA. This procedure yields time-frequency representations containing time-locked as well as non-time-locked responses. For this analysis, the present sample partially overlapped with the sample of 50 patients and 48 healthy controls described for which pre-training oscillatory activity in the paired-click design was described in [15].

Relevant time-frequency windows were defined using a cluster-based, independent-sample t-test with Monte Carlo randomization. This procedure effectively controls for multiple comparisons [52] and allows the identification of sensor clusters with significant group differences on a 3D sensor level (time, frequency, sensors). A cluster was determined to contain at least 5 neighboring sensors from 1000 randomizations for time-frequency data. The test statistic was defined as the sum of the t-statistics (T_sum_) within the respective 3D cluster. Empirically observed clusters are labeled as statistically significant if the probability of clusters gained from permutation being larger did not exceed 5%. Time windows and clusters were determined from the pre-training data. In addition to group differences in time-locked and non-time-locked activity measures prior to training, training effects on oscillatory activity parameters in the patient sample were evaluated in a repeated-measures ANOVA with the between-subjects factor Training group (CE vs. CP participants) and the within-subjects factor Time (pre- vs. post-training measures of evoked and induced activity). This ANOVA was based on the respective ROIs obtained after the cluster-based approach described above. In addition, effect sizes were evaluated using Hedges’g.

The relationships between spectral characteristics, M50 gating ratio, and performance variables were probed via Pearson correlations: for each individual the gating ratio was correlated with a set of power spectra that included every time–frequency bin for every MEG sensor. The resulting distributions of significant coefficients for distinct time–frequency bins were plotted as a function of sensor cluster, i.e., scalp topography.
